# Sepsis Due to Haemophilus influenzae and Masked Influenza A Infection

**DOI:** 10.7759/cureus.22424

**Published:** 2022-02-21

**Authors:** Sheri Walls, Prakash Adhikari, Hawwa Reesha, Martin Baxter

**Affiliations:** 1 Internal Medicine, Piedmont Athens Regional Medical Center, Athens, USA

**Keywords:** bacterial sepsis, influenza a infection, severe sepsis, gram-negative bacteremia, h. influenzae

## Abstract

*Haemophilus influenzae (H. influenzae)* is a facultative anaerobe, pleomorphic Gram-negative coccobacillus capable of causing various respiratory and blood stream infections. Introduction of childhood immunization against *H. influenza* type b has decreased its prevalence. Invasive infection with non-typeable *H. influenzae* is increasing specially in vulnerable population. We present a case of a 69-year-old female who developed septic shock due to* H. influenzae* infection. She was also found to have influenza A infection in bronchoalveolar lavage (BAL) sample although initial test with nasopharyngeal swab was negative. This case report highlights the fact that in patients with high clinical suspicion, negative nasopharyngeal swab with polymerase chain reaction may not rule out influenza infection and BAL may be necessary to confirm the diagnosis and* H. influenzae* can be causing bacterial superinfection in such patients. She was appropriately treated with third-generation cephalosporin for *H. influenzae* and with oseltamivir for influenza A. Her condition improved significantly with the treatment.

## Introduction

*Haemophilus influenzae* (*H. influenzae*) is a small, non-spore-forming coccobacillus classified into six serotypes (a-f) based on the capsule and non-typeable* H. influenzae* (NTHi) strains that lack a capsule. *H. influenzae* type b is the most virulent of all six serotypes but with widespread childhood immunization the prevalence has decreased and NTHi infection is more common in the United States. NTHi is a commensal microbe commonly found in upper and lower respiratory tract [1]. NTHi has multitude of virulence factors which provide resistance against complement and cause infections including bacteremia, meningitis, epiglottitis, and septic arthritis [2]. The infection is common in patients with neoplasm, asplenia, alcohol use disorder, human immunodeficiency virus infection, chronic pulmonary disease, long-term steroid use, or underlying viral lung infection. We present a patient with bacteremia due to *H. influenzae* and she was also found to have influenza A infection. The patient tested negative for influenza A by reverse transcriptase polymerase chain reaction (RT-PCR) with nasopharyngeal swab, but she was positive with bronchoalveolar lavage (BAL) sample.

## Case presentation

**Figure 1:**
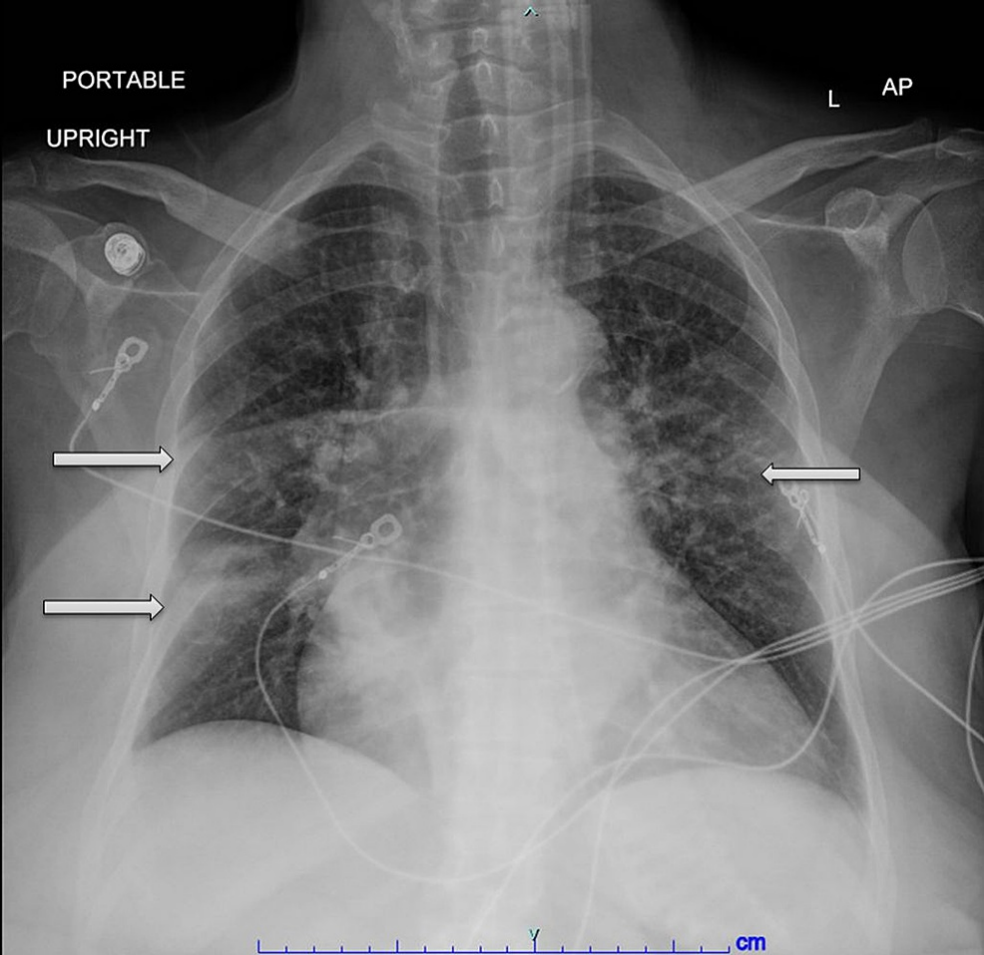
X-ray chest AP view; arrows show pulmonary opacities. AP, anteroposterior.

A 69-year-old female with a past medical history of atrial fibrillation, heart failure with reduced ejection fraction, hypertension, and chronic bilateral lymphedema presented to the emergency department with complaint of productive cough and shortness of breath for two days. She reported some associated fever, but temperature was not documented. She denied sick contact, recent travel, sputum production, wheezing, chest pain, diarrhea, or abdominal pain. She was up to date with vaccinations including flu shot and COVID-19 vaccination. On examination, her oxygen saturation was 82-83%, pulse rate 90 beats/minute, and blood pressure was normal. Her body mass index was 58 kg/m^2^. Lung examination showed bilateral decreased breath sounds with some inspiratory crackles. She had bilateral non-pitting edema on lower extremities suggestive of chronic lymphedema. Chest X-ray (Figure 1) showed bilateral infiltrates consistent with atypical pneumonia. Her blood test was significant for lactate of 3.8 mmol/l and normal electrolytes, and complete blood count showed normal white blood cell count with hemoglobin of 10.5 g/dl. COVID-19, influenza A, B, and respiratory syncytial virus with PCR were negative. Electrocardiogram showed atrial fibrillation with rapid ventricular response (121 beats/min). Her home medications included warfarin, iosartan, amlodipine, metoprolol, and digoxin. 

**Figure 2:**
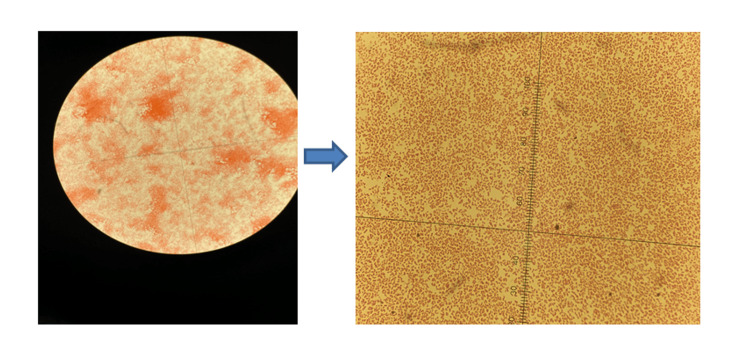
Microscopy showing Gram-negative bacilli (​​​​​​Haemophilus influenzae).

The patient was started on supplemental oxygen via nasal cannula, blood cultures were obtained, and vancomycin and piperracillin-tazobactem were initiated. Amiodarone infusion was started for rapid ventricular response. Her hospital course was complicated by worsening respiratory failure. She was transferred to intensive care unit (ICU), and she was intubated and put on mechanical ventilator. Her blood pressure was persistently low, and norepinephrine and epinephrine infusion was initiated. She also needed stress of dose steroid for refractory septic shock. Bronchoscopy was performed and the sample was sent for microscopy and culture. After 24 hours, blood culture isolated Gram-negative bacilli (Figure 2), which was identified as *H. influenzae*. The respiratory sample was sent for the pneumonia panel by PCR, which came positive for influenza A in addition to *H. * influenzae. Ceftriaxone and oseltamivir were started, and prior antibiotics were discontinued. Her condition gradually improved, and she was extubated on the seventh day. She was planned to receive 14 days of antibiotics for Gram-negative bacteremia and five days of oseltamivir.

## Discussion

*H. influenzae* is a facultative anaerobe, pleomorphic Gram-negative coccobacillus of Pasteurellaceae family. Among the subclass, *H. influenzae* type b is the most virulent [3]. *H. influenzae* conjugate vaccine (Hib) provides protection against capsular polysaccharide type b, and with the introduction of the childhood immunization in United States in 1987, *H. influenzae* type b infection has significantly decreased. In 2017, the incidence rate of *H. influenzae* type b was 0.18 per 100,000 population [3]. Now NTHi infection is increasing, causing majority of cases of otitis media, sinusitis, pneumonia, and bacteremia in Hib-immunized patients [4]. NTHi is the major cause of invasive infection in the geriatrics population and its incidence is around 6.2 per 100,000 population [3].* H. influenzae* are normal flora of upper respiratory tract and there are several mechanisms by which they adhere to the host cell and exhibit virulence. Encapsulated *H. influenzae* uses protein H and Haemophilus surface fibrils, whereas NTHi attaches directly to the surface epithelial cell, and it is resistant to the complement system [5].

Human influenza is an RNA virus of the Orthomyxoviridae family. They are classified into types A, B, and C based on nucleoprotein and matrix protein [4]. Influenza A virus is further subcategorized based on hemagglutinin and neuraminidase like H1N1, H1N2, and so forth. Gene mutations known as genetic drift are responsible for seasonal outbreak, whereas genetic shift is responsible for pandemics [6].

Pneumonia is one of the common systemic infections caused by *H. influenzae*. Presenting symptoms include high-grade fever, chills, lethargy, body aches, cough, chest pain, and shortness of breath [4,7]. It is indistinguishable from other bacterial pneumonia.* H. influenzae* pneumonia can be a bacterial superinfection in the background of viral infection or in the setting of immunocompromised state or sole infection. Our patient tested positive for Influenza A in a BAL sample. It was thought to be an inciting factor for invasive *H. influenzae* infection as she had no other contributing factor. 

Initial testing includes Gram stain, which shows pleomorphic Gram-negative coccobacilli. In our case, *H. influenzae* was identified by microscopy and culture but typing was not available in our facility. It was thought most likely to be NTHi as our patient was up to date with all childhood immunizations. Third-generation cephalosporin is the initial antibiotic of choice until culture and sensitivity results are available. The recommended duration of antibiotics is 10-14 days for Gram-negative bacteremia [3]. And for the diagnosis of influenza A, usually a real-time RT-PCR is performed in a nasopharyngeal swab sample. The sensitivity of the test is around 92% for influenza A and 100% for influenza B [8]. Our patient tested negative on a nasopharyngeal sample, but the BAL sample was positive for influenza A. The US Food and Drug Administration has approved neuraminidase inhibitors including oral oseltamivir and baloxavir, inhaled zanamivir, and intravenous peramivir for the treatment of influenza [9]. In most cases oral oseltamivir is the drug of choice. For better prognosis, treatment with neuraminidase inhibitors should be initiated as soon as possible and continued for five days [10]. Our patient recovered well with treatment with ceftriaxone and oseltamivir.

## Conclusions

Influenza A is a common cause of pneumonia in flu season. In patients with high clinical suspicion, negative nasopharyngeal swab with RT-PCR may not rule out infection and BAL may be needed to confirm diagnosis. Neuraminidase inhibitors should be initiated as soon as possible and continued for five days.* H. influenzae* is a facultative, Gram-negative coccobacillus, which can cause superinfection in cases of influenza viral pneumonia. NTHi is known for its invasive infection to cause Gram-negative bacteremia and sepsis. Third-generation cephalosporin is the initial antibiotic of choice for treatment.
